# Polymyxin E-Modified Conjugated Polymer Nanoparticle for Photodynamic and Photothermal Combined Antimicrobial Therapy

**DOI:** 10.3390/molecules31030409

**Published:** 2026-01-25

**Authors:** Qi Jiang, Yulu Hu, Huimin Ye, Xinyue Hu, Yue Yang, Minghui Yang, Fang Wang, Mengna Zhang, Lisheng Qian

**Affiliations:** College of Biomedical and Health, Anhui Science and Technology University, Chuzhou 233100, China

**Keywords:** polymyxin E, conjugated polymer nanoparticles, photodynamic antibacterial, photothermal antibacterial

## Abstract

The irrational or excessive use of antibiotics causes the emergence of bacterial resistance, making antibiotics less effective or ineffective. As the number of resistant antibiotics increases, it is crucial to develop new strategies and innovative approaches to potentiate the efficacy of existing antibiotics. Prior to this, we discovered that some of the traditional antibiotics produce reactive oxygen species (ROS) under specific light exposure. In this paper, we report a multifunctional polymeric nanoparticle (F8IC NPs-PME) that combines targeted and photodynamic–photothermal therapy (PDT-PTT) in one device. The PME on the surface of F8IC enables the selective binding of F8IC NPs-PME to the surface of Gram-negative bacteria. In addition, PME and F8IC can generate ROS and photothermia under near-infrared light excitation, respectively. The results showed that the sterilization efficiency of F8IC NPs-PME at a concentration of 8 μg/mL was as high as 94.7% against kanamycin-resistant *E. coli* under 808 nm near-infrared light irradiation (0.8 W/cm^2^, 10 min). This antimicrobial strategy can achieve efficient bacteria killing with a low dosage of antibiotics and opens up a new avenue for fighting bacterial resistance.

## 1. Introduction

Bacterial infection is one of the major factors affecting the rate of wound healing and has become the second leading cause of death worldwide [1,2]. As the most important antibacterial drugs, antibiotics have always provided a certain guarantee for human life and health [3,4]. In recent years, the inappropriate use or even abuse of antibiotics has led to an increasing number and variety of drug-resistant bacteria (Methicillin-resistant Staphylococcus aureus, Vancomycin-resistant Enterococcus, etc.), and the antimicrobial efficacy of some antibiotics has been declining or even ineffective [5,6,7,8]. Therefore, the development of new and effective antimicrobial methods and antimicrobial materials is urgent [9].

Light therapy, as an emerging therapy, is widely used in antimicrobial therapy due to its unique advantage of being less likely to induce drug resistance [10,11,12,13]. Photodynamic therapy (PDT) and photothermal therapy (PTT) have been widely used in bacterial killing as new phototherapeutic tools. And the antimicrobial treatment strategies of PDT and PTT based on nanomaterials are favored by the majority of researchers in the aspect of antimicrobial treatment [14,15]. Furthermore, it has been demonstrated that commonly encountered drug-resistant bacteria, including Methicillin-resistant *Staphylococcus aureus* and kanamycin-resistant *Escherichia coli*, exhibit comparable sensitivity to light therapy as non-resistant strains [16]. This finding highlights the promising potential of employing light therapy in combating drug-resistant bacterial infections. In recent years, many novel antimicrobial nanomaterials have been developed and successfully applied in antimicrobial therapy. Polymer nanoparticles have become one of the main research objects in the field of antimicrobial therapy because of their good biocompatibility, easy surface modification, and decorative nano-size effect [17,18]. Some polymer nanoparticles with good photothermal conversion efficiency have been applied to antitumor and antimicrobial therapy successfully.

4,4,10,10-tetrakis(4-hexylphenyl)-4,10-dihydrothieno [2″,3″:4′,5′]thieno [3′,2′:4,5]cyclopenta [1,2-b]thieno [2,3-d]thiophene-2,8-diyl)bis(2-(3-oxo-2,3-dihydroinden-5,6-difluoro-1-ylidene) malononitrile(F8-IC), as a type of conjugated polymer, has a good photothermal conversion efficiency. When the polymer was encapsulated with PEG to prepare water-soluble nanoparticles (F8-IC NPs), the photothermal conversion was as high as 82% [19]. The photothermal conversion efficiency of this nanoparticle is the highest compared to previously reported photothermal materials. In addition, the nanoparticles can be gradually degraded in the presence of biological enzymes and hydrogen peroxide, which greatly reduces the long-term cumulative toxicity of the nanomaterials in vivo, and thus the nanoparticles have a great potential for antimicrobial therapeutic applications [20]. Like most polymer nanoparticles, F8-IC NPs nanoparticles do not have the ability to target recognize bacteria. Additionally, overheating risks and limited tissue penetration depth are also major limitations. Therefore, it is worthwhile to investigate the use of F8-IC NPs for targeting bacteria to enhance antimicrobial efficacy.

Some narrow-spectrum conventional antibiotics have inherent properties of target binding to certain bacteria. For example, Vancomycin (Van) selectively binds Gram-positive bacteria and polymyxin E (PME) selectively binds Gram-negative bacteria [21,22]. Additionally, we observed that PME generates reactive oxygen species (ROS) when excited by 808 nm laser light. Therefore, the incorporation of PME can confer targeting properties to F8IC and effectively enhance its antibacterial efficacy through both targeting and its own ROS.

**Scheme 1:**
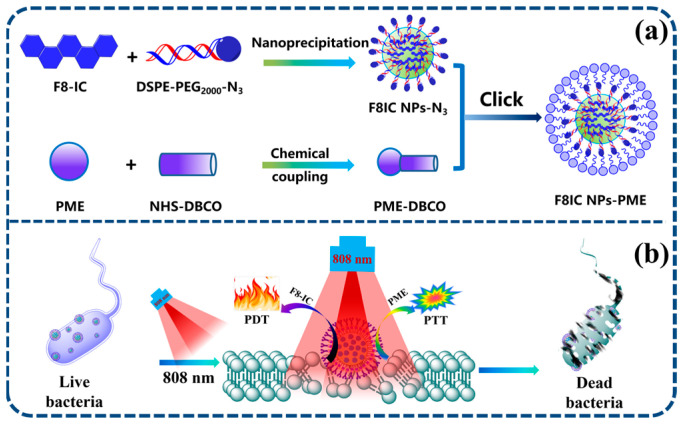
(**a**) Schematic diagram of the preparation of F8IC NPs-PME by click chemistry; (**b**) schematic diagram of the photodynamic and photothermal combined antimicrobial therapy of *E. coli* by F8IC NPs-PME.

**Scheme 2:**
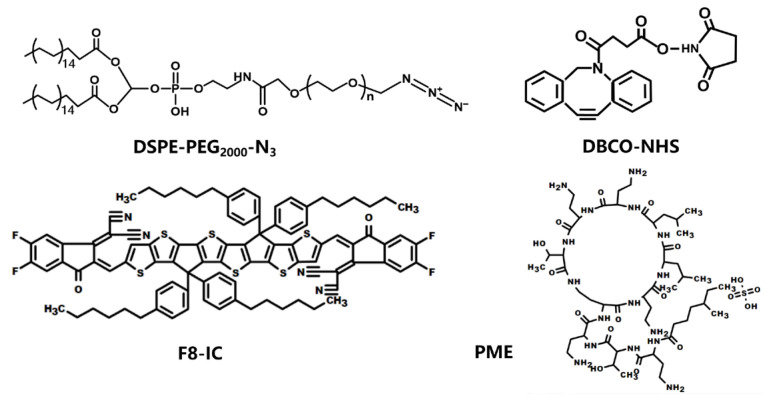
The structural formulae of DSPE-PEG_2000_-N_3_, DBCO-NHS, F8-IC, and PME.

In this study, we prepared a polymer nanoparticle integrating PDT and PTT (F8IC NPs-PME) based on the excellent photothermal conversion efficiency of F8IC NPs and the photodynamic activity of PME by nanoprecipitation method [23]. The F8IC NPs-PME was prepared by using a click chemistry reaction between azide-modified F8IC NPs and dibenzocyclooctyne (DBCO)-functionalized PME (Scheme 1). The structural formulae of the main reagents used are shown in Scheme 2. PME on the surface of F8IC NPs selectively binds F8IC NPs-PME to the surface of Gram-negative bacteria. In addition, PME and F8IC NPs can generate ROS and photothermia under the excitation of near-infrared light, which can be used for the combined antimicrobial treatment of Gram-negative bacteria with PDT-PTT. Therefore, F8IC NPs-PME nanoparticles enable efficient PDT-PTT combination antimicrobial therapy with precise targeting of Gram-negative bacteria. This antimicrobial strategy greatly broadens the application range of photosensitive antibiotics and opens up a new avenue for anti-infective therapy.

## 2. Results and Discussions

### 2.1. Characterization of F8IC NPs-PME Nanoparticles

**Figure 1:**
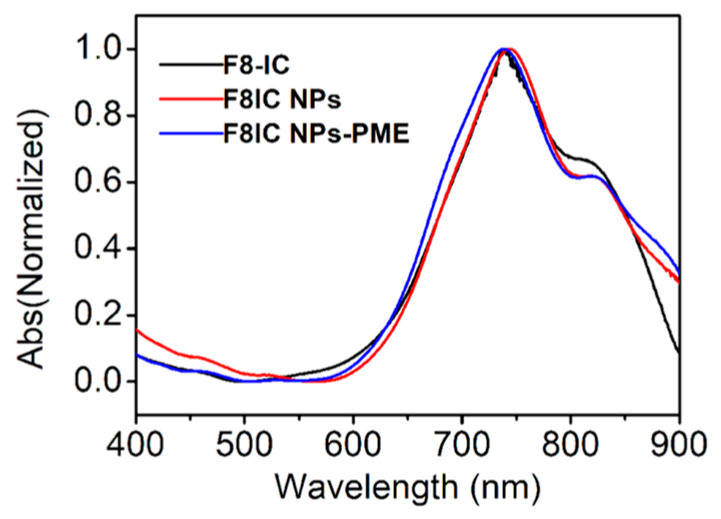
Normalized absorption spectrums of F8-IC, F8IC NPs, and F8IC NPs-PME aqueous dispersion.

Figure 1 shows the UV-Vis absorption spectra of F8-IC, F8IC NPs, and F8IC NPs-PME. Both F8-IC, F8IC NPs, and F8IC NPs-PME have the strongest absorption peak at 735 nm. This result shows that the preparation of nanoparticles and the modification of PME did not affect the optical properties of F8-IC. It is also evident from the spectra that the nanoparticles have strong absorption in the near-infrared region, which also indicates the potential and advantages of F8IC NPs-PME in photothermal antimicrobial therapy.

**Figure 2:**
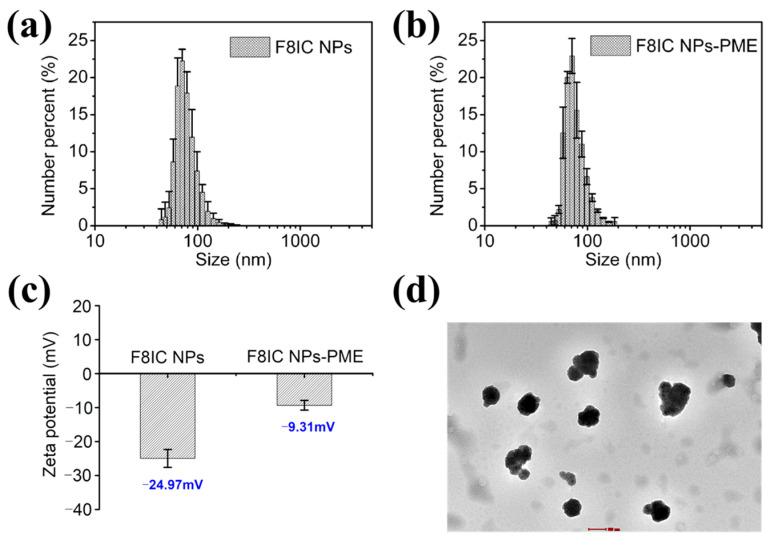
Characterization of particle size, zeta potential, and morphology of F8IC NPs and F8IC NPs-PME. DLS particle size maps of F8IC NPs (**a**) and F8IC NPs-PME (**b**). (**c**) Zeta potential of F8IC NPs and F8IC NPs-PME. (**d**) TEM pictures of F8IC NPs-PME. Scale bar is 100 nm. Standard deviations were obtained from three parallel experiments.

We characterized the morphology of F8IC NPs-PME nanoparticles using TEM. As shown in Figure 2d, the F8IC NPs-PME nanoparticle is a spherical-like nanoparticle with a diameter of about 80 nm. We further characterized the hydrated particle size of F8IC NPs and F8IC NPs-PME using DLS (Figure 2a,b). The DLS results showed that the hydrated particle size of F8IC NPs and F8IC NPs-PME nanoparticles was about 75 nm, which was consistent with the TEM characterization results. However, the TEM image indicates that the nanoparticles exist as conglomerates. In subsequent research, we will incorporate stabilizers or surface modification techniques to attempt isolation of individual particles, to determine whether this approach can enhance their associated properties.

In addition, we characterized the zeta potential of F8IC NPs and F8IC NPs-PME nanoparticles using DLS. As shown in Figure 2c, the zeta potential value of F8IC NPs is −24.97 mV, while that of F8IC NPs-PME is −9.31 mV. The higher zeta potential value of F8IC NPs-PME relative to F8IC NPs suggests that PME was successfully modified on the surface of F8IC NPs. Because PME itself is positively charged, the modification of PME leads to an increase in the positive charge on the surface of F8IC NPs and an increase in the zeta potential.

### 2.2. Exploration of F8IC NPs-PME Nanoparticles’ Specific Recognition of E. coli

**Figure 3:**
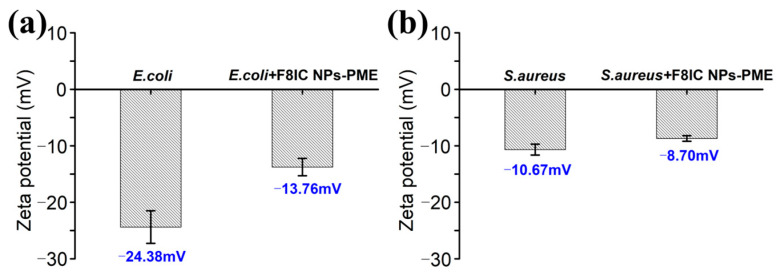
Zeta potential of (**a**) *E. coli* and (**b**) *S. aureus* with or without F8IC-PME targeting.

To explore the functions of specific recognition and fluorescence imaging of F8IC NPs-PME nanoparticles, we chose *E. coli* and *S. aureus* as model bacteria. Figure 3a shows the zeta potentials of naked and F8IC NPs-PME-treated bacteria. The zeta potentials of the *E. coli* group and the *E. coli* + F8IC NPs-PME group were −24.38 mV and −13.76 mV, respectively. When F8IC NPs-PME binds to the surface of *E. coli*, it causes an increase in the zeta potential of *E. coli*. Because the potential of F8IC NPs-PME (−9.31 mV) is higher than that of the *E. coli*. As shown in Figure 3b, Zeta potentials of *S. aureus* alone (−10.67 mV) and *S. aureus* teeated with F8IC NPs-PME (−8.70 mV) were comparable. This result suggests that F8IC NPs-PME nanoparticles can specifically bind Gram-negative bacteria.

### 2.3. Investigating ROS and Photothermal Properties of F8IC NPs-PME Nanoparticles

**Figure 4:**
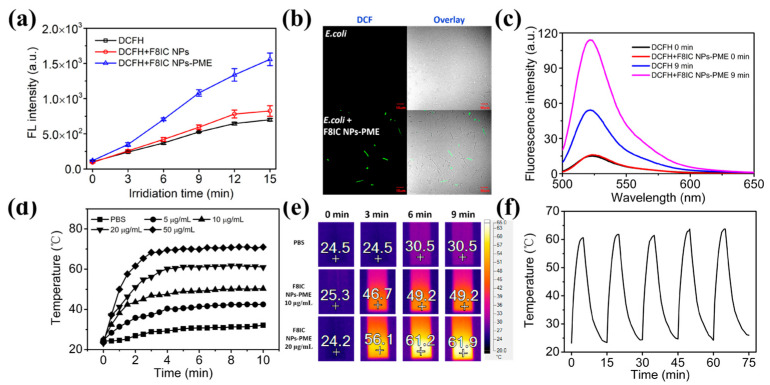
Characterization of ROS and photothermal conversion properties of F8IC NPs-PME. (**a**) Fluorescence signal values of DCF at 525 nm in solutions with different light exposure times (*n* = 3); (**b**) CLSM of ROS generation by F8IC NPs-PME on *E.coli* Surface (scale:10 μm); (**c**) DCF fluorescence spectroscopy; (**d**) temperature variation in F8IC NPs-PME solutions with different concentrations over time; (**e**) photothermal imaging of F8IC NPs-PME under different illumination times; (**f**) temperature profile of F8IC NPs-PME solution with laser on or off for 5 times.

The fluorescence dye, 2′,7′-dichlorodihydrofluorescein diacetate (DCF-DA), is nonfluorescent and becomes fluorescent as 2′,7′-dichlorodihydrofluorescein (DCF) nonspecifically in the presence of a variety of ROS [24]. We explored the ROS-generating ability of F8IC NPs-PME nanoparticles using DCFH-DA. As shown in Figure 4a, with the increase in light time, the DCF fluorescence signal in the F8IC NPs nanoparticle solution remained basically the same as that in the blank group, while the DCF fluorescence signal in the F8IC NPs-PME solution was significantly higher than that in the blank group. This result indicates that F8-IC NPs-PME can produce ROS under the excitation of 808 nm laser (1.5 W cm^−2^). In addition, we utilized CLSM fluorescence imaging to further validate that F8IC NPs-PME bound to *E. coli* is also capable of producing ROS. From Figure 4b, in the absence of F8IC NPs-PME, DCF fluorescence was almost invisible in *E. coli* in vivo after exposure to light. In contrast, *E. coli* combined with F8IC NPs-PME showed obvious DCF green fluorescence visible in vivo after light exposure. This result suggests that F8IC NPs-PME has the potential to photodynamically kill *E. coli*.

We have explored the photothermal properties of F8IC NPs-PME nanoparticles. The light source was an 808 nm laser with a light intensity of 1.0 W/cm^2^. As shown in Figure 4d, the temperatures of F8IC NPs-PME nanoparticle solutions of different concentrations all increased rapidly with the increase in light time. The temperature of F8IC NPs-PME nanoparticle solution increased rapidly in 0–5 min of light exposure, and basically leveled off after 5 min. It can also be seen from the figure that as the concentration of the material increases, the temperature of the solution also increases. When the concentration of F8IC NPs-PME was 20 μg/mL, the temperature of the solution rose to about 62 °C after 5 min of light exposure. This temperature was sufficient to rupture the cell membrane of the bacteria, which eventually led to the death of the bacteria. Good photothermal stability is demonstrated if the temperature of the material is stable over five photoperiods. We subjected the F8IC NPs-PME solution to five consecutive 15 min heating and cooling cycles and did not observe any significant degradation of the photothermal properties until the end of the cycle (Figure 4f). The material is shown to have excellent photothermal stability under 808 nm laser light.

### 2.4. Antibacterial Investigation of F8IC NPs-Ab and F8IC NPs-PME Against E. coli

**Figure 5:**
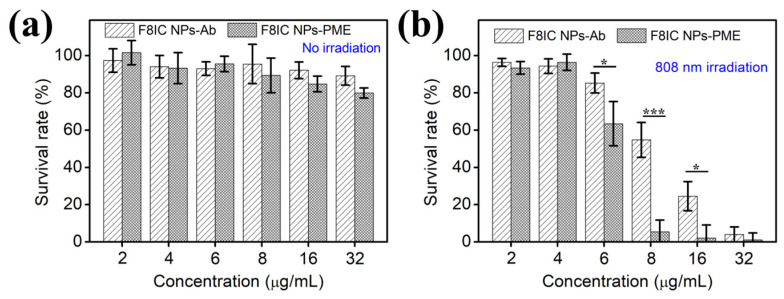
The viability of *E. coli* treated by F8IC NPs-Ab and F8IC NPs-PME without light-irradiation (**a**), and with the same light-irradiation (1.0 W cm^−2^, 10 min) (**b**). (* *p* < 0.05, *** *p* < 0.001).

To validate the combined antibacterial effect of F8IC NPs-PME, we selected an antibody-modified F8IC NPs (F8IC NPs-Ab) that can specifically bind to *E. coli* and compared its killing effect with F8IC NPs-PME. The viability of *E. coli* that is treated with F8IC NPs-Ab and F8IC NPs-PME in the range of 2–32 μg/mL remains intact without irradiation (Figure 5a). The survival rate of *E. coli* under irradiation gradually decreases with the increase in F8IC NPs-Ab and F8IC NPs-PME concentration from 2 to 32 μg/mL. When exposed to light irradiation, *E. coli* treated with 8 μg/mL F8IC NPs-PME exhibits a survival rate of merely 5.7%. This is substantially lower than the survival rate for F8IC NPs-Ab (54.8%), as clearly shown in Figure 5b. It follows that the antimicrobial effect comes not only from the heat generated by F8IC NPs, but also from the ROS generated by PME. In addition, it depends on the method of preparation of F8IC NPs-PME, without taking into account the loss of PME, where the maximum content of PME in F8IC NPs-PME was 0.24 μg/mL.

**Figure 6:**
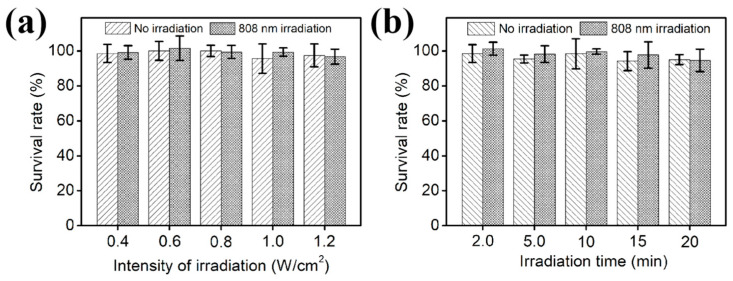
Effect of different irradiation intensities (**a**) and irradiation durations (**b**) of separate 808 nm sources on *E. coli* activity.

In order to avoid the interference of the light source itself on the experiment, we investigated the effect of different light duration and light intensity of the light source on the bacteria (Figure 6). As the light intensity increased from 0.4 to 1.2 W cm^−2^, the activity of *E. coli* basically remained above 90%. Similarly, *E. coli* activity remained above 90% with increasing irradiation time from 2 to 20 min. Therefore, choosing light intensity and light duration within this range during the subsequent experiments can avoid the influence of the light source itself on the experimental results.

**Figure 7:**
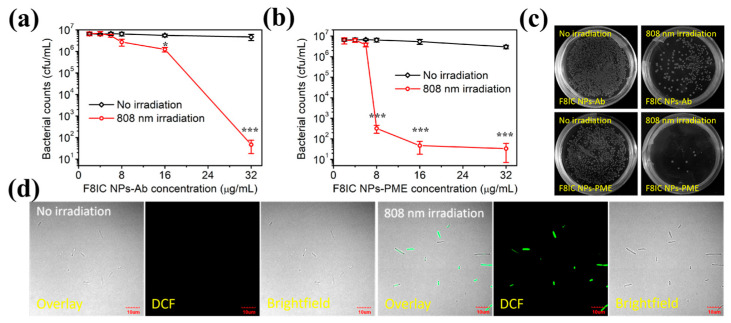
Antibacterial effect of F8IC NPs-Ab (**a**) and F8IC NPs-PME (**b**). (**c**) Plate photographs for *E. coli* diluted by the same times. (**d**) CLSM of *E. coli* stained by SYTOX green. The concentration of both F8IC NPs-Ab and F8IC NPs-PME are 8 μg/mL. (* *p* < 0.05; *** *p* < 0.001).

In addition, we explored the antimicrobial effects of different concentrations of F8IC NPs-Ab and F8IC NPs-PME using colony counting method. As shown in Figure 7a,b, under the optimal light conditions, the activity of *E. coli* gradually decreased when the F8IC NPs-Ab concentration was increased from 2 μg/mL to 32 μg/mL. When the concentration of F8IC NPs-Ab was increased to 32 μg/mL, the number of colonies in the illuminated group decreased by about 5.01 orders of magnitude compared with that in the unilluminated group. When the concentration of F8IC NPs-PME was increased from 2 μg/mL to 32 μg/mL, the activity of *E. coli* in the light group also decreased significantly, while the activity of *E. coli* in the unlighted group remained basically unchanged. However, when the concentration of F8IC NPs-PME reached 16 μg/mL, the colony number of the light-illuminated group decreased by about 5.06 orders of magnitude compared with that of the non-light-illuminated group. From the figure, it can be seen that the amount of F8IC NPs-PME is lower than that of F8IC NPs-Ab for the same antimicrobial effect. This result proves once again that F8IC NPs-PME has a highly efficient combined photodynamic–photothermal antimicrobial effect.

To further demonstrate the combined photodynamic–photothermal antimicrobial effect of F8IC NPs-PME, we stained F8IC NPs-PME-treated *E. coli* with STYOX green fluorescent dye, and then used CLSM fluorescence imaging to observe the survival of *E. coli*. SYTOX green is a nucleic acid fluorescent dye that penetrates easily through damaged cell membranes but not through normal cell membranes, and is therefore commonly used to verify cell membrane disruption. As shown in Figure 7d, the green fluorescence of SYTOX green was hardly observed in *E. coli* in the unilluminated group, while the green fluorescence of SYTOX green was obvious in the field of view of *E. coli* in the illuminated group.

### 2.5. Validation of F8IC NPs-PME Specificity for Photodynamic–Photothermal Killing of E. coli

**Figure 8:**
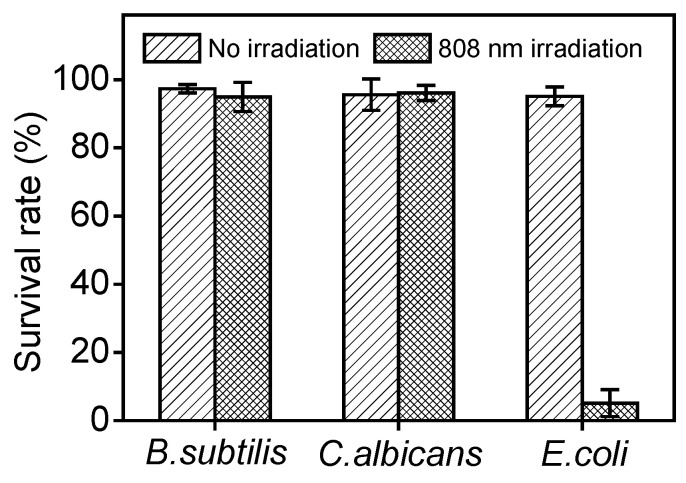
Validation of the combined photodynamic–photothermal antimicrobial specificity of F8IC NPs-PME against *E. coli*.

In order to verify the specificity of F8IC NPs-PME for killing *E. coli*, we compared the antimicrobial efficiency of F8IC NPs-PME on Gram-negative bacteria (*E. coli*), Gram-positive bacteria (*B.subtilis*), and fungi (*C. albicans*). As shown in Figure 8, under the same concentration and light conditions (8 μg/mL, 0.8 W/cm^2^, 10 min), the survival rate of *S. aureus* and *C. albicans* in both the unlit and light groups was above 90%. In contrast, the survival rate of *E. coli* in the light group was only about 5%. This result indicates that F8IC NPs-PME has excellent selective photodynamic–photothermal antimicrobial effect against *E. coli*, which may provide a new way for specific targeted photodynamic–photothermal combined killing of *E. coli*.

## 3. Materials and Methods

### 3.1. Materials

The amphiphilic substances (DSPE-PEG_2000_-N_3_) and the coupling agents (DBCO-NHS) were obtained from ShangHai ToYangBio Tech. Inc., Shanghai, China. The polymer monomer (F8-IC) was purchased from derthon Optoelectronic Materials Science Technology Co., Ltd., Shenzhen, China. 2,7-Dichlorofluorescein diethyl ester (DCFH-DA) and Polymyxin E sulfate (PME) were sourced from Beijing Solare Technology Co., Ltd., Beijing, China. Dimethyl sulfoxide (DMSO), tetrahydrofuran (THF, 99.5%), and glutaraldehyde were purchased from J&K. Kanamycin-resistant *E. coli* (BL21) and *S. aureus* (ATCC 6538) were provided by China General Microbiological Culture Collection Center.

The UV-vis spectra of nanoparticles and the optical densities of bacteria were measured using a UV-2600 UV-vis spectrophotometer (Shimadzu, Kyoto, Japan) and a Microplate Reader Multiskan GO (Thermo Scientific, Waltham, MA, USA), respectively. Fluorescence spectra of the solution were detected on a Hitachi F-4600 spectrofluorometer (Tokyo, Japan) equipped with a xenon lamp. CLSM experiments were performed with a FV1000-IX81 confocal laser scanning microscope (Olympus, Tokyo, Japan). Light irradiation tests were performed with the 808 nm laser (Changchun New Industries Optoelectronics Technology Co., Ltd., Changchun, China). Data processing and plotting software includes Origin (Origin 8.5), Photoshop (Adobe Photoshaop CS5), SmartView and Office (Office2013).

### 3.2. Methods

Synthesis and functionalized modification of F8IC NPs. F8IC NPs were synthesized using the nanoprecipitation method [24]. A mixture containing 250 μL of F8-IC (1 mg/mL) and 100 μL of DSPE-PEG_2000_-N_3_ (1 mg/mL) was added to 6500 μL of THF and sonicated for 5 min. This solution was then introduced dropwise at a rate of 10 μL/min into 9 mL of water under continuous stirring. After complete addition, stirring was continued for 30 min. Subsequently, the mixture was heated in a 90 °C water bath under a nitrogen atmosphere to evaporate THF. To remove aggregates formed during synthesis, the resulting F8IC NPs were filtered through a 0.22 μm microporous membrane. Further purification and concentration were performed using a 100 kDa molecular weight cutoff ultrafiltration membrane. The concentrated nanoparticle suspension was stored at 4 °C.

F8IC NPs-PME was prepared by conjugating azide-functionalized F8IC NPs with the amine groups of PME via DBCO-NHS crosslinking. Briefly, 50 μL of PME (1 mg/mL) and 2 μL of DBCO-NHS (5 mg/mL) were combined with 948 μL of 1 × PBS and incubated with shaking for 30 min. Then, 500 μL of F8IC NPs (40 μg/mL) was added, and the reaction mixture was shaken at 4 °C for 2 h. The conjugate was purified using a 100 kDa centrifugal filter and diluted to a concentration of 40 μg/mL before storage at 4 °C. F8IC NPs-Ab was prepared following a similar procedure.

Characterization of morphology, particle size, and Zeta potential by TEM and DLS. For TEM imaging, the F8IC NPs-PME sample was diluted and ultrasonically dispersed for 20 min. The nanoparticles were then negatively stained with 10 μL of 2% phosphotungstic acid, followed by three washes with water. Finally, the grid was dried using a vacuum freeze dryer. And the morphology was characterized by TEM (Tecnai G2 F20 S-TWIN, FEI, Hillsboro, OR, USA). For DLS measurements, 1 mL of each diluted sample was analyzed to measure the hydrodynamic diameter and zeta potential by DLS (ZEN3700, Malvern, UK).

Investigating reactive oxygen species (ROS) generation of F8IC NPs-PME. ROS detection in vitro: First, 20 μL F8IC NPs-PME and 40 μL DCFH were added to 140 μL 1 × PBS. The final concentrations of F8IC NPs-PME and DCFH were 4 μg/mL and 8 μg/mL, respectively. All samples were irradiated with an 808 nm laser (1.0 W/cm^2^) for varying durations (0, 3, 6, 9, 12, and 15 min). After irradiation, the DCF fluorescence signal between 500 and 700 nm (excitation at 480 nm) was measured using an F-4600 fluorimeter (Hitachi, Tokyo, Japan). Each condition was tested in triplicate.

ROS detection in vivo: Following mixing, 800 µL of *E. coli* suspension was combined with 2 µL of DCFH-DA and 198 µL of LB medium, and the final concentration of DCFH-DA was 0.02 mmol/L. The mixture was then incubated at 37 °C with shaking at 180 rpm for 20 min. Subsequently, the solution was centrifuged to remove excess DCFH-DA, followed by two washes with PBS. The bacteria were resuspended in LB medium. In total, 800 μL bacteria, 100 μL 1 × PBS, and 100 μL F8IC NPs-PME (20 μg/mL) were taken into a centrifuge tube and incubated at 37 °C, 80 rpm for 20 min in a shaking table. After 10 min of illumination under a 808 nm laser (1.0 mW cm^−2^), the bacteria solution was centrifuged at 8000 rpm for 3 min and the supernatant was removed. Then 200 μL of 0.5% glutaraldehyde aqueous solution was used to resuspend the bacteria and wash once by centrifugation after fixation for 30 min at room temperature. Finally, the pellet with 20 μL 1 × PBS was resuspended and 10 μL for CLSM (488 nm excitation) was taken to observe the fluorescence of DCF.

Investigating photothermal properties of F8IC NPs-PME. To assess the photothermal effect, an 808 nm laser was applied to 500 μL aliquots of F8-IC NPs-PME at different concentrations (0, 5, 10, 20, 50, and 100 μg/mL). An infrared thermal imager (Ti450, FLUKE) was used to record the sample temperature at 10 min intervals.

Minimum inhibitory concentration (MIC) of F8-IC NPs-PME and F8-IC NPs-Ab. The bacterial suspension at 0.8 OD was first diluted 40-fold. Subsequently, 80 µL aliquots of the diluted suspension (approximately 2 × 10^6^ CFU per well in LB medium) were dispensed into a 96-well plate. Different concentrations of F8IC NPs-PME (or F8IC NPs-Ab) were then added to each well, and the total volume was brought to 100 µL with additional LB medium. Following addition, the plates were exposed to a white light source for varying durations. Finally, they were incubated overnight (approximately 16 h) at 37 °C with shaking at 180 rpm.

Finally, the bacterial killing efficiency was determined by calculating the survival rate (%) with the following formula: Survival Rate (%) = [(C − B)/(A − B)] × 100%. Here, A represents the absorbance of the control (bacteria without nanomaterials), B is the absorbance of the background (LB medium alone), and C is the absorbance of the sample (bacteria treated with varying concentrations of nanomaterials).

Antibacterial activity test by colony counting. *E. coli* cultures were grown in LB medium at 37 °C with shaking (180 rpm) until reaching an OD_600_ of approximately 0.8. The culture was then diluted 40-fold, and 800 µL aliquots were dispensed into a 48-well plate. Different concentrations of PMB-CON were added to each well, bringing the final volume to 1 mL. Four experimental groups were tested: (1) bacteria with F8IC NPs (control), (2) bacteria with F8IC NPs under 808 nm irradiation (1.0 W/cm^2^, 10 min), (3) bacteria with F8IC NPs-PME, and (4) bacteria with F8IC NPs-PME under 808 nm irradiation (1.0 W/cm^2^, 10 min). After treatment, plates coated under different conditions were incubated at 37 °C, and CFUs was counted.

Antibacterial effect of PMB-CON by SYTOX Green dye. To assess membrane integrity, 800 μL of *E. coli* was treated with F8IC NPs-PME (8 μg/mL) or PBS (control) in 1 × PBS, followed by shaking (800 rpm, 37 °C, 10 min) and 808 nm irradiation (1.0 W/cm^2^, 10 min). SYTOX Green (1 mmol/L) was then added, and incubation continued (800 rpm, 37 °C, 20 min). After two PBS washes, cells were fixed in 0.5% glutaraldehyde (30 min) and washed by PBS. And 10 μL suspension was imaged via CLSM to visualize SYTOX Green fluorescence.

## 4. Conclusions

In summary, a multifunctional antibacterial nanoparticle, F8IC NPs-PME, was synthesized by conjugating the photosensitive antibiotic PME to F8IC via click chemistry. Compared with other antibacterial nanoparticles, the F8IC NPs-PME system offers several distinct advantages: First, leveraging PME’s specific affinity for Gram-negative bacteria, F8IC NPs-PME enables targeted identification and binding of *E. coli*. Second, through the combined photodynamic activity of PME and the photothermal properties of F8IC, the system achieves synergistic photodynamic/photothermal killing of kanamycin-resistant *E. coli*. Thus, F8IC NPs-PME integrates targeted recognition, PDT, and PTT into a single platform. This multifunctional approach enhances the efficiency of killing drug-resistant Gram-negative bacteria while reducing the required dosage of PME. Overall, this photosensitive antibiotic modification strategy broadens the utility of conventional antibiotics and offers a promising avenue for combating bacterial resistance.

## Data Availability

The original contributions presented in the study are included in the article/Supplementary Materials, further inquiries can be directed at the corresponding authors.

## Supplementary Materials

The following supporting information can be downloaded at https://www.mdpi.com/article/10.3390/molecules31030409/s1, Figure S1: DCF fluorescence spectroscopy of PME solution; Figure S2: The viability of *E. coli* treated by F8IC NPs in dark and light (1.0 W cm^−2^, 10 min); Figure S3: The zeta potential(a) and particle size(b) of F8IC NPs-PME using DLS under different storage times.
