# Supplementary material for: Polymyxin E-Modified Conjugated Polymer Nanoparticle for Photodynamic and Photothermal Combined Antimicrobial Therapy
Source: Molecules. 2026 Jan 25;31(3):409. doi: 10.3390/molecules31030409 (PMC12898741; doi:10.3390/molecules31030409)
Supplement: Supplementary file 1 [file molecules-31-00409-s001.zip › molecules-4007660-supplementary.pdf]

Supplementary Information (SI) for Molecules.

Supplementary Information

Polymyxin E-modified conjugated polymer nanoparticle for photodynamic and photothermal combined antimicrobial therapy

Qi Jiang [1,*], Yulu Hu [1], Huimin Ye [1], Xinyue Hu [1], Yue Yang [1], Minghui Yang [1], Fang Wang [1], Mengna Zhang [1], Lisheng Qian [1,*]

College of Biomedical and Health, Anhui Science and Technology University, Chuzhou 233100, China;

*Corresponding author

E-mail: jiangqi@ahstu.edu.cn (Q.J.); qianls@ahstu.edu.cn (L.Q.)

Table of Contents

1. Characterization of Reactive Oxygen Species（ROS） in PME

2. The antibacterial activity of F8IC NPs

3. The zeta potential and particle size of F8IC NPs-PME using DLS under different storage times

1. Characterization of Reactive Oxygen Species（ROS） in PME

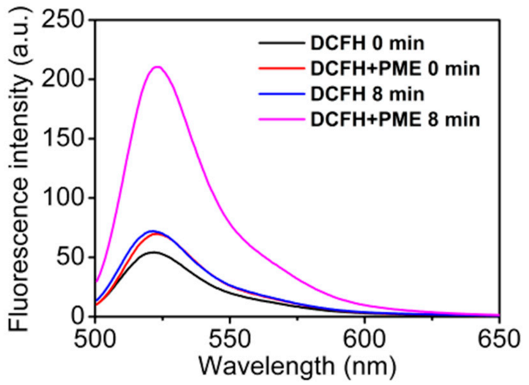

Figure S1. DCF fluorescence spectroscopy of PME solution.

We employed DCFH to detect the total ROS production of PME upon excitation by an 808 nm light source. As shown in Figure S1, DCF fluorescence at 525 nm was barely detectable in PME solution under dark conditions. However, under 808 nm light illumination(1.5 W cm^{-2}), strong DCF fluorescence signals were observed in samples. This result indicates that PME can generate ROS under specific light conditions, demonstrating photodynamic activity.

2. The antibacterial activity of F8IC NPs

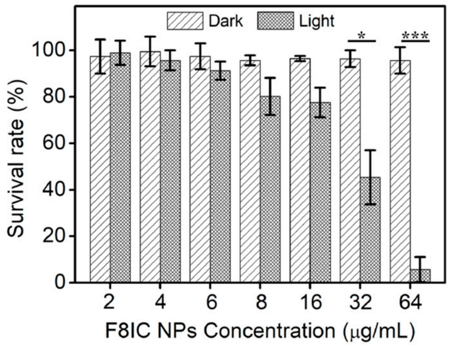

Figure S2. The viability of *E. coli* treated by F8IC NPs in dark and light (1.0 W cm^{-2}, 10 min).

(*$p < 0.05$, ***$p < 0.001$).

To further demonstrate the targeted efficacy of PME, we investigated the antibacterial effect of F8IC NPs(Figure S2). Compared with Figure 7 in the manuscript, F8IC NPs-PME exhibits higher antibacterial efficiency. This result, serving as a non-specific nanoparticle control, further demonstrates that PME possesses a certain degree of targeting effect.

3. The zeta potential and particle size of F8IC NPs-PME using DLS under different storage times

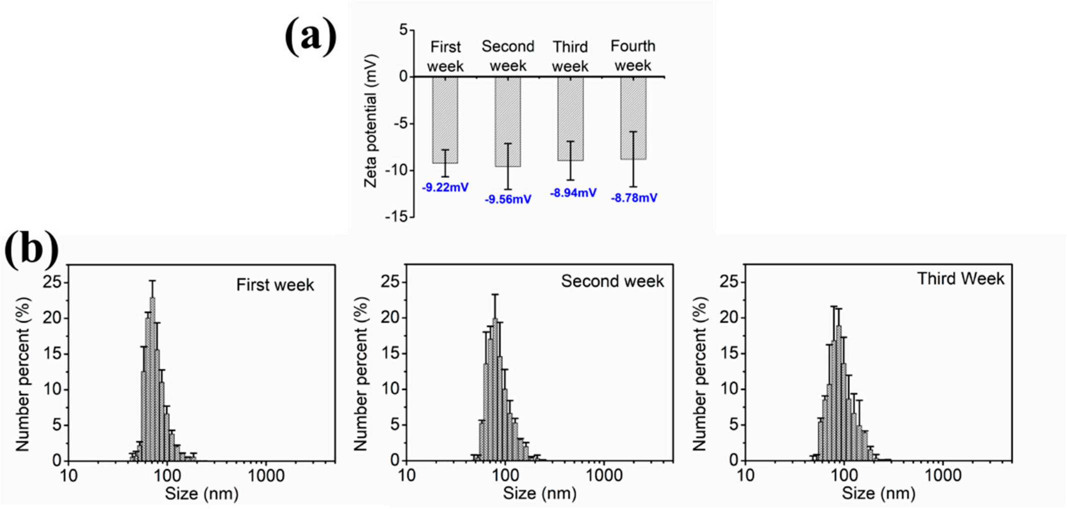

Figure S3. The zeta potential(a) and particle size(b) of F8IC NPs-PME using DLS under different storage times.

To investigate the stability of F8IC NPs-PME, we analyzed their zeta potential and particle size by DLS after storage for different durations. As shown in the figure S3, the zeta potential and particle size of F8IC NPs-PME remained virtually unchanged over the four-week period. This result indicates that the nanoparticles exhibit excellent stability over a short timeframe. Of course, as time increases, the degree of particle size dispersion of the F8IC NPs-PME increases(Figure S3b), indicating that the nanoparticles exhibits aggregation over time.
